# The diagnostic yield of nasopharyngeal aspirate for pediatric pulmonary tuberculosis: a systematic review and meta-analysis

**DOI:** 10.1186/s44263-023-00018-1

**Published:** 2023-10-18

**Authors:** Nisreen Khambati, Rinn Song, Emily Lai-Ho MacLean, Mikashmi Kohli, Laura Olbrich, Else Margreet Bijker

**Affiliations:** 1 Oxford Vaccine Group, Department of Pediatrics, University of Oxford, Oxford, UK.; 2 Faculty of Medicine and Health, Central Clinical School, The University of Sydney, Sydney, Australia.; 3 Centre of Research Excellence in Tuberculosis, Sydney, NSW, Australia.; 4 Foundation for Innovative New Diagnostics, Geneva, Switzerland.; 5 Division of Infectious Diseases and Tropical Medicine, University Hospital, Munich, Germany.; 6 German Centre for Infection Research (DZIF), Partner Site Munich, Munich, Germany.; 7 Fraunhofer Institute ITMP, Immunology, Infection and Pandemic Research, Munich, Germany.; 8 Department of Pediatrics, Maastricht University Medical Center, MosaKids Children’s Hospital, Maastricht, the Netherlands.

**Keywords:** Tuberculosis, Nasopharyngeal aspirate, Diagnosis, Diagnostic yield, Children, Systematic review, Meta-analysis

## Abstract

**Background:**

Tuberculosis (TB) is a leading cause of death in children, but many cases are never diagnosed. Microbiological diagnosis of pulmonary TB is challenging in young children who cannot spontaneously expectorate sputum. Nasopharyngeal aspirates (NPA) may be more easily collected than gastric aspirate and induced sputum and can be obtained on demand, unlike stool. However, further information on its diagnostic yield is needed.

**Methods:**

We systematically reviewed and meta-analyzed the diagnostic yield of one NPA for testing by either culture or nucleic acid amplification testing (NAAT) to detect *Mycobacterium tuberculosis* from children. We searched three bibliographic databases and two trial registers up to 24th November 2022. Studies that reported the proportion of children diagnosed by NPA compared to a microbiological reference standard (MRS) were eligible. Culture and/or WHO-endorsed NAAT on at least one respiratory specimen served as the MRS. We also estimated the incremental yield of two NPA samples compared to one and summarized operational aspects of NPA collection and processing. Univariate random-effect meta-analyses were performed to calculate pooled diagnostic yield estimates.

**Results:**

From 1483 citations, 54 were selected for full-text review, and nine were included. Based on six studies including 256 children with microbiologically confirmed TB, the diagnostic yield of NAAT on one NPA ranged from 31 to 60% (summary estimate 44%, 95% *CI* 36–51%). From seven studies including 242 children with confirmed TB, the diagnostic yield of culture was 17–88% (summary estimate 58%, 95% *CI* 42–73%). Testing a second NPA increased the yield by 8–19% for NAAT and 4–35% for culture. NPA collection procedures varied between studies, although most children had NPA successfully obtained (96–100%), with a low rate of indeterminate results (< 5%). Data on NPA acceptability and specifically for children under 5 years were limited.

**Conclusions:**

NPA is a suitable and feasible specimen for diagnosing pediatric TB. The high rates of successful collection across different levels of healthcare improve access to microbiological testing, supporting its inclusion in diagnostic algorithms for TB, especially if sampling is repeated. Future research into the acceptability of NPA and how to standardize collection to optimize diagnostic yield is needed.

## Background

Tuberculosis (TB) is a leading cause of death in children [1]. Calculating accurate mortality rates in children is difficult since many cases are never diagnosed or reported [2, 3]. Microbiological diagnosis of TB enables confirmation of disease and initiation of appropriate treatment, including treatment for drug-resistant TB when indicated, through detection of resistance to antimicrobial agents. However, this is challenging in children because they often have paucibacillary disease, and most young children cannot voluntarily produce good quality sputum specimens, the standard sample collected in adults [4]. Underdiagnosis is therefore common, with most pediatric TB deaths occurring in those who did not receive treatment [5].

The World Health Organization (WHO) has recommended induced sputum, gastric aspirate (GA), stool, and nasopharyngeal aspirate (NPA) as alternative samples for diagnosing pediatric TB [6]. Sputum induction requires electricity and equipment for the nebulization [7] and a well-ventilated area with adequate infection control measures to mitigate the transmission risk [8]. Overnight fasting is needed for good-quality GA samples, often necessitating hospital admission [6]. Sputum induction and gastric aspiration can thus be challenging to implement at lower-level health facilities due to operational and resource limitations, including adequately trained staff [9]. Whilst stool collection is non-invasive, stool can rarely be passed on demand, and there is a potential for invalid results or errors using molecular detection techniques [10].

Nasopharyngeal aspiration involves inserting a small catheter into the nasopharynx to stimulate a cough reflex, with aspiration of secretions into a mucus trap [11]. It does not require hospital admission like GA and has fewer transmission risks than the collection of induced sputum [7]. Although trained personnel and equipment are still needed, results from a large randomized trial found that 97% of children with symptoms of pneumonia had an NPA successfully obtained. In comparison, only 81% of children had stool collected [12]. NPA collection has the potential to be implemented across varying levels of the healthcare system, thereby increasing access to TB diagnosis. However, further information on its diagnostic yield using existing TB diagnostic tools is needed.

We conducted a systematic review and meta-analysis on detecting *Mycobacterium tuberculosis* (Mtb) using culture or nucleic acid amplification testing (NAAT) on NPA from children evaluated for pulmonary TB (PTB). Our primary aim was to estimate the proportion of children diagnosed by NPA compared to a microbiological reference standard (MRS) and, where available, compared to a composite or clinical reference standard (CRS). As secondary aims, we estimated the incremental yield of two NPA samples compared to one and summarized information on operational aspects of NPA collection and processing. To our knowledge, this is the first systematic review focusing on both the diagnostic yield and operational aspects of NPA for pediatric TB.

## Methods

This systematic review was reported according to the PRISMA diagnostic test accuracy (DTA) guidelines [13]. The PRISMA checklist is available in Additional file 1.

### Protocol and registration

The protocol for this systematic review is registered at PROSPERO — CRD42021283965 (https://www.crd.york.ac.uk/prospero/display_record.php?RecordID=283965).

### Search strategy

We conducted a systematic search of PubMed, Embase, and the Cochrane Library published up to 24th November 2022, with no other time limits. The search strategy was constructed with a medical librarian and incorporated text words and database subject headings related to the index specimen — “nasopharyngeal aspirate” and the target condition — “tuberculosis.” Complete search strategies for each database are presented in the supplementary material (Additional file 2). We also checked reference lists of included studies and review articles. For unpublished or ongoing studies, we searched ClinicalTrials.gov and the WHO International Clinical Trials Registry Platform and contacted study authors when potentially eligible unpublished studies were identified.

### Eligibility criteria

We included studies that reported the number of participants under 18 years with presumed PTB and the number that was diagnosed using culture or NAAT on NPA in comparison to an appropriate MRS, irrespective of HIV status, previous TB testing, or anti-TB treatment of any duration. Original data studies written in English, French, Italian, Portuguese, German, and Dutch, utilizing any study design or enrolment timing and evaluating fresh or banked specimens, were eligible. We excluded conference proceedings, editorials, reviews, and studies using mixed adult and pediatric populations, unless they reported accuracy results for children separately. We also excluded studies if data were available only on a per-specimen basis rather than on a per-child basis, which we deemed more meaningful for clinical practice, where usually multiple tests and sample types per child are used to diagnose TB.

### Study screening and selection

After removing duplicates, two reviewers (N. K. and E. B.) independently screened titles and abstracts per eligibility criteria, followed by full-text review for inclusion in the systematic review. Any disagreement was resolved through discussion with a third reviewer (LO).

### Data extraction

We designed an Excel data extraction form and piloted it on two studies, after which the form was optimized and used for all selected full-text articles. Two reviewers (N. K. and E. B.) independently extracted data for the diagnostic yield of NPA culture or NAAT compared to the MRS as defined below and, where available, a CRS. We also collected information on study characteristics and population and data on NPA sample collection and processing for a post hoc analysis on operational aspects of NPA. Disagreements were discussed until consensus was reached. We contacted study investigators regarding missing data and clarification and stratification of diagnostic performance, if needed.

### Quality assessment

Two reviewers (N. K. and E. B.) independently assessed the methodological quality of included studies using the quality assessment of diagnostic accuracy studies-2 (QUADAS-2) framework [14]. The adapted tool was first piloted with two studies. Discrepancies were resolved by discussion between NK and EB, with a third reviewer (L. O.) consulted if needed. The QUADAS-2 tool with signaling questions tailored to this review and justification for assigning levels of bias is included in the supplementary material (Additional file 3).

### Reference standards

We defined the MRS as mycobacterial culture and/or a WHO-endorsed NAAT on any clinical specimen for diagnosing childhood PTB, including induced sputum, GA, NPA, stool, string test, expectorated sputum, and bronchoalveolar lavage as per international case definitions for pediatric intrathoracic TB [15]. Children who were MRS positive were defined as having confirmed TB. WHO-endorsed NAATs include Xpert MTB/RIF (Xpert) and Xpert MTB/RIF Ultra (Ultra) (Cepheid, USA), Truenat MTB (Molbio, India), and moderate complexity automated NAATs [16]. Since inclusion of positive TB cases by NPA in the MRS could overestimate the diagnostic yield, we also defined a modified MRS where NPA was not included. We anticipated definitions of the CRS to be heterogeneous across studies and used the definitions in original publications. CRS in studies included children with confirmed TB and children with clinically diagnosed TB based on symptoms and signs, radiological changes, exposure history, immunological evidence, and treatment response (unconfirmed TB) [15].

### Data synthesis and statistical analysis

For the primary objective, we calculated the diagnostic yield of NPA with 95% confidence intervals (CI) for individual studies. We defined this as the proportion of children diagnosed with PTB using either culture or NAAT on NPA compared to the number of children positive by MRS (confirmed TB) and, where available, to the number of children positive by CRS (confirmed + unconfirmed TB). Diagnostic yield was based on one NPA sample. In studies evaluating multiple NPA specimens, the first NPA sample was used. Secondarily, to assess the incremental yield of a second NPA specimen versus the MRS, we included studies where data could be extracted for both the first and second NPA samples.

We performed meta-analyses to estimate the pooled diagnostic yield for culture or NAAT on one NPA with univariate random-effect hierarchical models. All studies were included irrespective of the risk of bias. In a pre-specified sensitivity analysis, we calculated the pooled diagnostic yield after excluding studies with a high or unclear risk of bias for the reference standard. This was used as a proxy for the quality of the study. Observed proportions from individual studies were transformed to a natural logarithm scale to account for skewed data and extreme proportions. Results from individual studies and summary estimates were demonstrated in forest plots, with the *I*^2^statistic (95% CI) used to quantify between-study heterogeneity. To explore sources of heterogeneity, we conducted sub-analyses stratified by HIV status and age. All analyses were conducted using the “metafor” and “meta” packages in R version 4.2.2 [17, 18].

## Results

### Search results

**Fig. 1:**
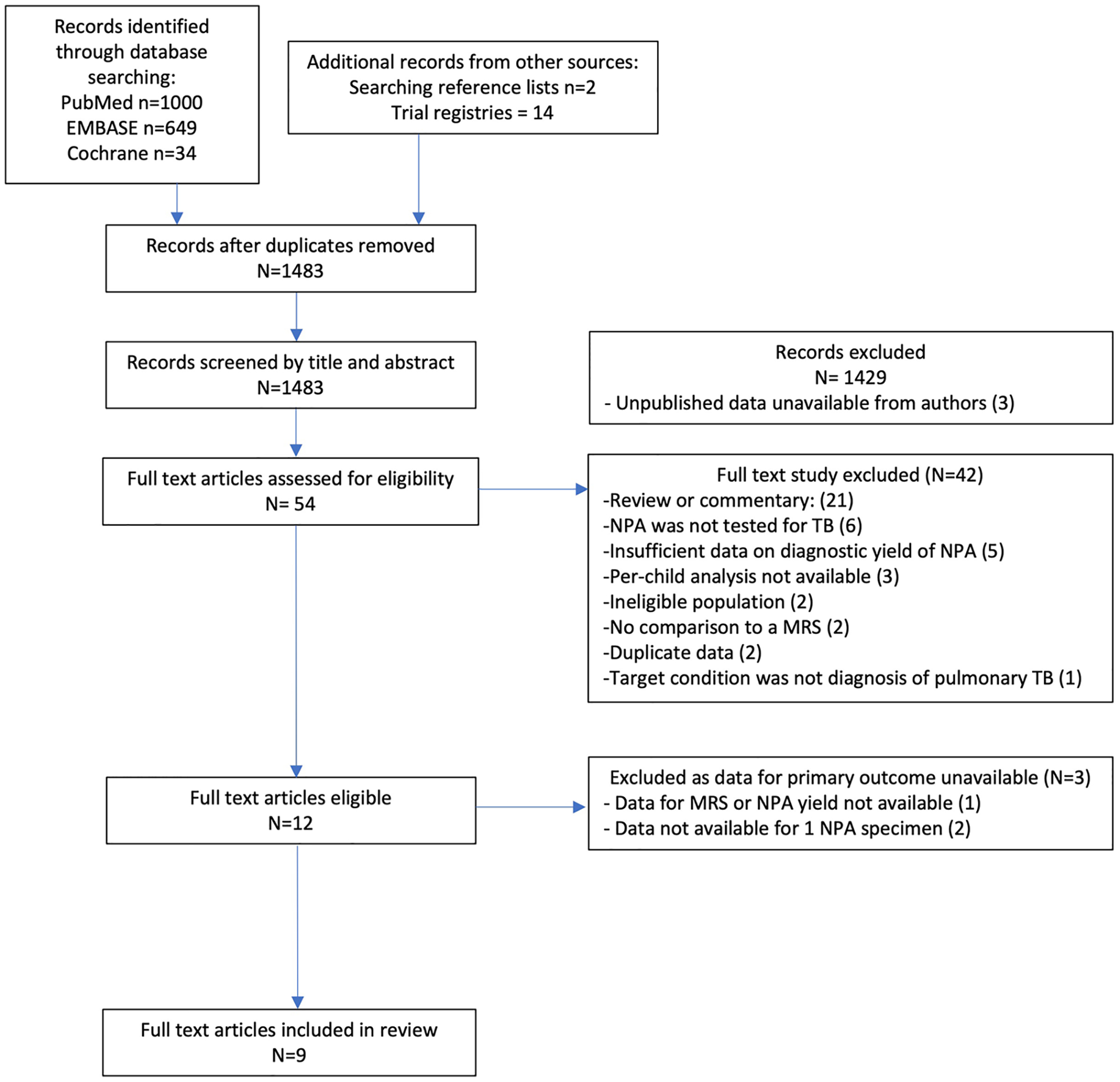
PRISMA study flow diagram

We identified 1483 unique studies, of which 54 were selected for full-text review and 12 met our eligibility criteria (Fig. 1). We identified three unpublished studies (NCT04121026, NCT04240990, NCT04038632) for which data were unavailable from the authors. Three eligible studies were excluded because data for our primary objective were unavailable despite contacting authors. Specifically, two studies only reported the combined NPA diagnostic yield based on two samples [19, 20]; one stopped NPA collection during the study, and data extraction on NPA yield or the MRS was not possible [21]. The remaining nine studies were included in this systematic review.

### Study and patient characteristics

Study and patient characteristics are presented in Table 1. Participants were recruited across eight high-TB burden countries, mostly within Africa, with 7/9 studies including cohorts with a high HIV prevalence as per WHO definition [22]. Seven studies recruited in hospitals, with one additionally recruiting from the community [23], and two solely from primary-level health facilities [24, 25]. The most common exclusion criterion was current or previously received TB treatment within varying time periods. The prevalence of children positive by MRS (confirmed TB) varied widely, ranging from 3 [24] to 41% [11]. The prevalence of children positive by CRS (confirmed and unconfirmed TB) ranged from 51 [25] to 90% [26]. Additional file 4: Table S1 summarizes the MRS and CRS definitions of the included studies.

NPA collection, processing, and applied microbiological tests varied between studies (Table 2). The proportion of children with one NPA collected was high, ranging from 96 to 100% [11, 24, 26, 28], although collection rates for two samples across consecutive days were slightly lower (83/105, 79%) [24]. Operational aspects that might affect diagnostic yield were not uniformly reported. The target volume of NPA was only described in one study (2–5 ml) [26], and no study reported the actual volume collected. The proportion of uninterpretable NPA NAAT results was less than 5% [24–26]. Only one study reported the total proportion of contaminated NPA cultures (31/184, 17%) [24]. The culture method differed across studies, including liquid culture: mycobacteria growth indicator tube (MGIT) 960 or microscopic observation drug susceptibility (MODS) and solid culture: Löwenstein–Jensen or 7H11. For NAAT, most studies used Xpert (5/9), with the remaining using Ultra (1/9), the real-time RealArt^™^ PCR kit (1/9), or in-house heminested PCRs (2/9). For studies that tested both NAAT and culture [11, 23, 24, 26–29], NPA specimens were split for separate testing.

### Quality assessment

**Fig. 2:**
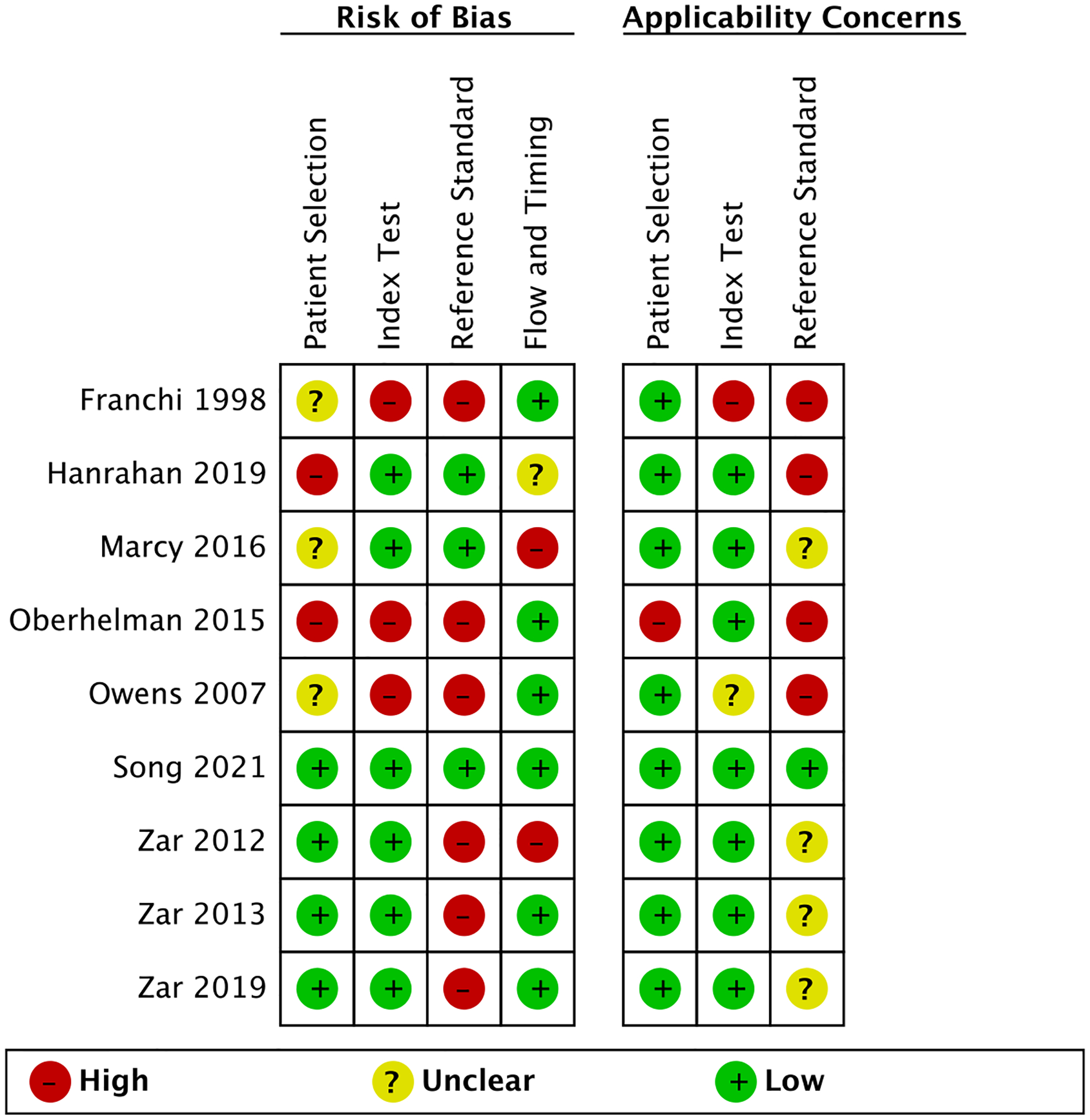
Summary of risk of bias and applicability concerns using QUADAS-2 tool. The review authors’ judgements about each domain are presented for each included study

**Fig. 3:**
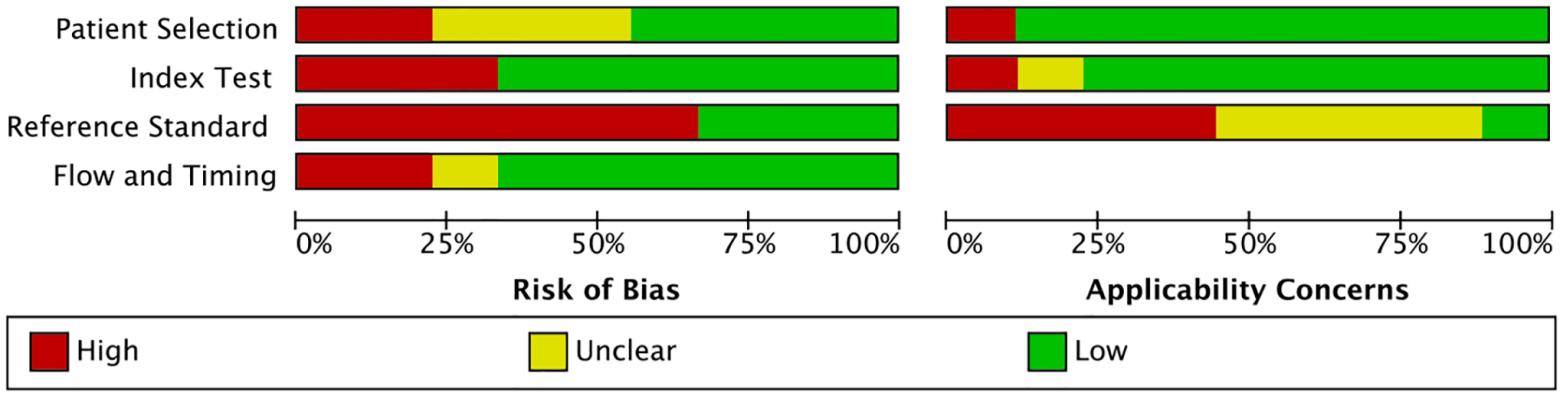
Summary of risk of bias and applicability concerns using QUADAS-2 tool. The review authors’ judgements about each domain are presented as percentages across the included studies

Figures 2 and 3, Additional file 5: Table S2 summarize the QUADAS-2 assessments. Seven out of nine studies had a low or unclear risk of bias (ROB) for patient selection. Two had a high ROB for excluding the clinically unwell and children above 10 years [24] and comparing cases to healthy controls [27]. Applicability concerns were overall low, except for one case–control study which enrolled asymptomatic children with a positive tuberculin skin test, a test not routinely used for TB screening in most high-burden settings [27].

For the index test domain, most included studies had a low ROB since they used tests with automatically generated results and pre-specified thresholds (Xpert, Ultra, and MGIT). Most studies reported an appropriate method of mucus extraction with suction, so applicability concerns were overall low, except for two studies [11, 28].

The reference standard domain scored most poorly. We only scored three studies as having a low ROB since they collected multiple different specimens and used both culture and a WHO-endorsed NAAT [23, 24, 26]. Applicability concerns were high in four studies for not reporting specification methods to distinguish Mtb from other mycobacteria [11, 24, 27, 28].

Most studies had a low ROB for the flow and timings domain. One study included substantially fewer children in the analyses than the number enrolled (loss of 20%) [29]. In another, children received different culture reference tests [26], known to have differing sensitivities [31]. Both were scored as having a high ROB.

### Diagnostic yield of NPA

**Fig. 4:**
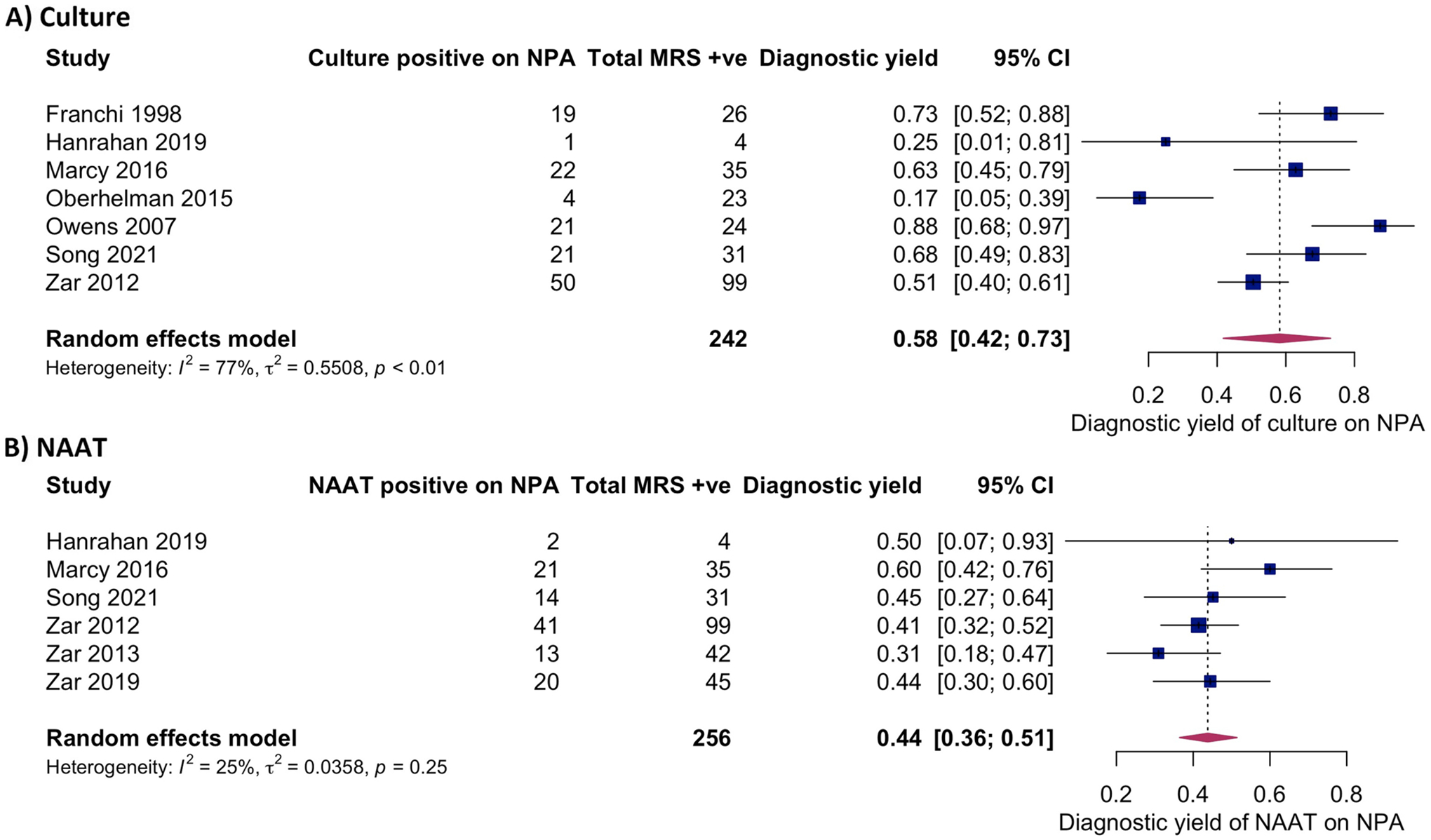
NPA diagnostic yield compared to children positive for MRS, according to study test. **A** Culture on NPA. **B** NAAT on NPA

Seven studies (including 242 children with confirmed TB) evaluated the diagnostic yield of culture on one NPA against the MRS [11, 23, 24, 26–29]. A total of 17 to 88% of children with confirmed TB were diagnosed using culture on NPA (Fig. 4, Additional file 6: Table S3). The pooled estimate was 58% (95% *CI* 42–73%). Nonoverlapping CIs between some studies and an *I*^2^ value of 77% (95% *CI* 57–98%) indicated considerable between-study heterogeneity.

The diagnostic yield of NAAT on one NPA versus the MRS could not be extracted in three studies which used in-house PCRs or the RealArt^™^ PCR kit [11, 27, 28]. In the remaining six studies (including 256 children with confirmed TB), 31 to 60% of children with confirmed TB were diagnosed using NAAT on one NPA (Fig. 4, Additional file 6: Table S3) [23–26, 29, 30]. The pooled estimate was 44% (95% *CI* 36–51%) with CIs largely overlapping. The *I*^2^ value was 25% (95% *CI* 0–88%).

We calculated diagnostic yield of NPA, excluding NPA in the MRS (modified MRS) (Additional file 7: Table S4). This data was only available from 2/6 studies for NAAT and 5/7 studies for culture. Diagnostic yield relative to this modified MRS was very similar compared to diagnostic yield relative to the original MRS, except for one study with very small numbers of children with TB [24].

Based on three studies with data available against a CRS, 1 to 15% of children with confirmed and unconfirmed TB were diagnosed using culture on one NPA [24, 26, 29]. Based on five studies, 2 to 14% of children with confirmed and unconfirmed TB were diagnosed using NAAT on one NPA [24–26, 29, 30] (Additional file 8: Table S5). Given the small number of studies and the significant heterogeneity observed in CRS definitions, meta-analyses were not done.

**Fig. 5:**
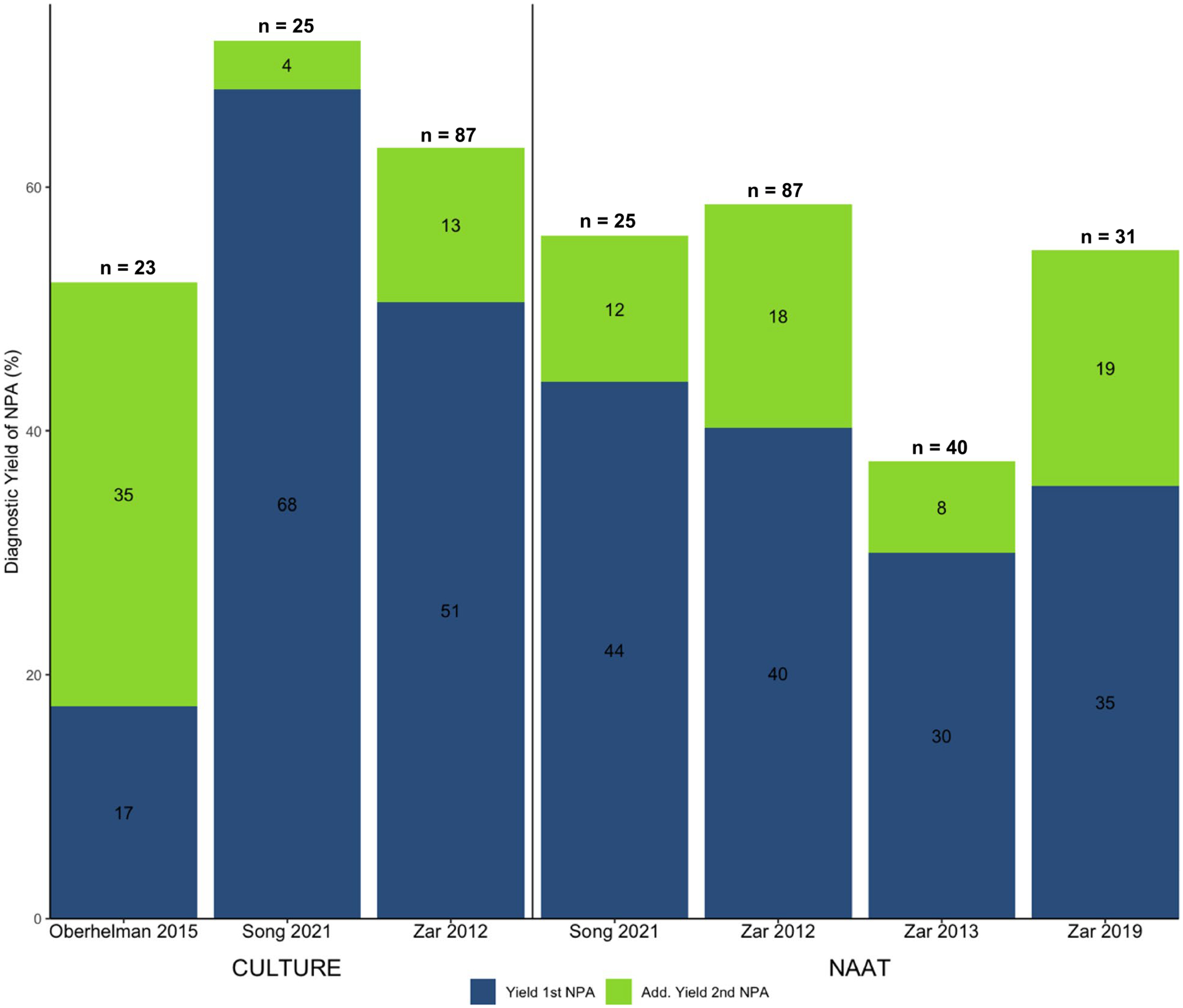
Incremental diagnostic yield of a second NPA using culture or NAAT compared to a MRS. The number in bars refers to the diagnostic yield of either the 1st or 2nd NPA sample in %. *n* refers to the total number of children with microbiologically confirmed TB in each study (MRS positive)

Testing two NPA samples compared to single sample testing increased the diagnostic yield by 4–35% for culture [23, 27, 29] and by 8–19% for NAAT [23, 25, 29, 30] versus a MRS (Fig. 5). The percentage of children with microbiologically confirmed TB by testing of other specimens who were not detected by two NPAs varied from 28 to 48% for culture and 41–63% for NAAT.

We undertook two sensitivity analyses for the meta-analyses (Additional file 9: Table S6). Firstly, we only included the three studies with a low ROB for the MRS [23, 24, 26]. Pooled diagnostic yield for culture (63%, 95% *CI* 51–74%) and NAAT (53%, 95% *CI*41–64%) were similar to the pooled estimates from all the studies. Secondly, three studies in the main analyses for culture on NPA did not include both NAAT and culture in the MRS [11, 27, 28]. A MRS that only includes one reference test may detect fewer confirmed cases in the denominator for diagnostic yield, which could lead to overestimation of the result. To address this, these three studies were post hoc excluded, which did not change the pooled estimate (57%, 95% *CI* 46–68%) compared to our main analyses. *I*^2^ values were lower in both sensitivity analyses suggesting less heterogeneity.

We also explored study heterogeneity based on HIV status and age. There were too few studies to derive pooled estimates for subgroups and conduct meta-regression; however, inspection of forest plots suggested a higher trend in NPA yield among CLHIV than HIV-negative children for culture and NAAT (Additional file 10: Fig. S1) and a higher trend in diagnostic yield in younger children for NAAT (Additional file 10: Fig. S2). Since diagnostic yield of NPA is influenced by the number of MRS-positive children in a study, we also conducted a post hoc analysis to explore this. Visual inspection of scatterplots suggested a positive relationship between microbiological confirmation rate and NPA culture diagnostic yield (Additional file 10: Fig. S3). This association was not observed for NAAT (Additional file 10: Fig. S3).

## Discussion

In this systematic review and meta-analysis, microbiological testing of one NPA specimen detected Mtb in approximately half of all children with microbiologically confirmed TB. The summary diagnostic yield of culture (58%; 95% *CI* 42–73%) was slightly higher than the summary estimate of Xpert or Xpert Ultra (44%; 95% 36–51%). Whilst we confirmed the added value of repeated NPA samples to increase microbiological yield by 4–35% for culture and 8–19% for NAAT, two samples will, at best, still miss a third of children with TB.

We identified between-study heterogeneity in NPA diagnostic yield, especially for culture. Whereas all studies in our meta-analysis for NAAT used the GeneXpert Systems, culture methods varied. Liquid culture is more sensitive than solid culture [32], and using both improves Mtb recovery if contamination occurs [33]. Differences in the reference standard likely contributed to heterogeneity, reflected in the lower *I*^2^ in the sensitivity analyses only including studies with a low ROB for the MRS and studies with two reference tests as opposed to one, although this should be interpreted with caution given the few studies and the wide *I*^2^95% CIs [34]. Diagnostic yield also depends on the quality and volume of the specimen. The minimum volume for NPA recommended by the WHO is 2 ml, although larger volumes are associated with greater bacteriological yield [35]. Limited data on NPA volumes and other aspects of the collection process made it difficult to evaluate the impact on yield.

Heterogeneity in yield can be due to variation in study population and the pre-test probability of TB. Indeed, the microbiological confirmation rate, which is highly influenced by the patient population, appeared to be related to the yield for culture on NPA. Patients were recruited from different levels of healthcare facilities, with tertiary referral centers more likely to have children with advanced disease and higher mycobacterial burdens [36]. The trend for a higher NPA yield in CLHIV compared to HIV-negative children suggested in our review has been noted in other diagnostic specimens [37–39] and could be related to the greater risk of TB and more advanced disease. In contrast, the trend for a greater NPA yield in younger children is surprising since they often have paucibacillary disease, although other factors may contribute to these findings.

Operational factors including feasibility and acceptability influence the choice of specimen collection [6]. The high proportion of children with successful NPA collection (> 95%) across different levels of healthcare and the low rate of indeterminate results with NAAT (< 5%) in our review support the feasibility of NPA. This is consistent with the recent TB-speed pneumonia study where 97% (1140/1169) of children with symptoms of pneumonia across six high TB incidence countries had NPA collected, and only 1.3% (15/1132) of Ultra results on NPA were invalid or had errors, although this study only recruited from hospitals [12]. No study in our review provided data on the acceptability of NPA. Preliminary findings from a cross-sectional qualitative study within the TB-speed pneumonia project identified that whilst NPA collection was perceived as painful by nurses and parents, it was overall well-accepted and judged to be quicker and less invasive than GA [40].

Our diagnostic yield estimates for NPA were lower than sensitivity estimates for Xpert Ultra on expectorated or induced sputum (75.3%), GA (70.4%), and stool (56.1%) based on a reference standard of culture in another meta-analysis for pediatric TB [38]. However, the use of different MRS definitions hampers this comparison, and indirect comparison of specimens between studies can be biased by differences in population and setting. Although testing of NPA will miss some children with TB, detection is significantly improved when a combination of different samples is utilized [26]. Obtaining different specimens in 1 day may be simpler than collecting samples over consecutive days. In a study of children with presumptive TB in South Africa, a combination of one induced sputum and NPA using Ultra identified 80% of children with confirmed TB [30]. Similarly, in a pediatric study in Kenya, testing one NPA and stool with MGIT and Xpert had a diagnostic yield of 71%, which was comparable to the yield from two GA (77%) over multiple days [23]. NPA, as a relatively easy procedure, can increase access to microbiological testing, with yield improved if feasible by testing additional specimens.

There are several strengths to our review. We conducted a search strategy that covered six languages and included the three key bibliographic databases recommended for diagnostic studies [41] and trial registers for unpublished studies. Although our inclusion criteria were limited to European languages, we did not find any article that could not be screened due to language restrictions. Most studies in our review included CLHIV and had an average age of under 5 years, suggesting our results are highly applicable to key diagnostic groups. Our dataset included children from different levels of health facilities across three continents, improving the generalizability of our findings. We conducted multiple sensitivity analyses to check our assumptions and explore alternative explanations for our findings. Finally, we considered diagnostic yield estimates separately for NAAT and culture. Access to culture is restricted to highly specialized health facilities [42], whereas automated NAAT has lower technical and infrastructure requirements and is more suitable for lower-level health settings [7]. Distinguishing these two tests reflects their different potential roles in TB diagnostic algorithms.

This review and evidence base do have limitations. Whilst pooled estimates can summarize information across multiple studies, between-study heterogeneity, especially for culture, means that they must be interpreted with caution, and readers are encouraged to consider the variety in yield estimates as shown in the forest plots. Although we performed sub-analyses based on HIV and age, paucity of data meant we could not conduct meta-regression to fully explore how these variables contributed to differences in NPA diagnostic yield. Secondly, we included NPA in the MRS, which can potentially overestimate the diagnostic yield. However, diagnostic yield was very similar for nearly all studies when using a modified microbiological reference standard in which NPA results were excluded [15]. Thirdly, whereas all studies using NAAT on NPA had culture and NAAT in their MRS, some studies only had culture in their MRS, potentially skewing estimates. However, our sensitivity analysis showed minimal change to diagnostic yield. Finally, despite contacting authors, we had to exclude three eligible studies as data for our primary aim could not be extracted.

Whilst the feasibility of NPA supports decentralization to lower levels of healthcare, we identified several gaps in the evidence to be addressed. Firstly, more qualitative research is needed on the perspectives of children, caregivers, and health workers on NPA, especially regarding acceptability, repeated sampling, and barriers to collection. Although reporting was incomplete, we noted variation between studies in many aspects of NPA collection. Protocols for NPA themselves are not uniform; whereas the WHO suggests 2 h of fasting prior to NPA collection [6], other national and international bodies do not [43–45]. Operational research into standardizing and optimizing sample processing and collection in low-resource settings to enhance recovery of bacilli from NPA is recommended. Finally, improved reporting on the performance of NPA specifically for children under 5 could help researchers better understand its value where it is most clinically relevant.

## Conclusions

Our systematic review and meta-analysis confirm the suitability of NPA as an alternate specimen for the microbiological confirmation of pediatric PTB. Despite suboptimal diagnostic yield, the high rates of successful collection across different levels of healthcare help improve access to microbiological testing. This supports the inclusion of NPA in diagnostic algorithms for TB, especially if sampling is repeated or in combination with other specimens.

## Supplementary Material

PRISMA checklist. **Additional file 1.** PRISMA checklist.

Search strategy for systematic review and meta-analysis for each database. **Additional file 2.** Search strategy for systematic review and meta-analysis for each database.

Adapted QUADAS-2 tool. **Additional file 3.** Adapted QUADAS-2 tool.

MRS as defined by our review and CRS definitions across the studies. **Additional file 4: Table S1.** MRS as defined by our review and CRS definitions across the studies.

Detailed summary of QUADAS-2 assessments for each study. **Additional file 5: Table S2.** Detailed summary of QUADAS-2 assessments for each study.

Diagnostic yield for NPA culture and NPA NAAT compared to children positive for MRS. **Additional file 6: Table S3.** Diagnostic yield for NPA culture and NPA NAAT compared to children positive for MRS.

Diagnostic yield for NPA culture and NPA NAAT compared to a modified MRS. **Additional file 7: Table S4.** Diagnostic yield for NPA culture and NPA NAAT compared to a modified MRS.

Diagnostic yield for NPA culture and NPA NAAT compared to children positive for CRS. **Additional file 8: Table S5.** Diagnostic yield for NPA culture and NPA NAAT compared to children positive for CRS.

Summary estimates for diagnostic yield from main and sensitivity analyses, compared to MRS. **Additional file 9: Table S6.** Summary estimates for diagnostic yield from main and sensitivity analyses, compared to MRS.

Fig. S1. NPA diagnostic yield compared to children positive for MRS, according to HIV status. Fig. S2. NPA diagnostic yield using NAAT compared to children positive for MRS, according to age. Fig. S3. Scatterplot between the study microbiological confirmation rate and the diagnostic yield of NPA. **Additional file 10: Fig. S1.** NPA diagnostic yield compared to children positive for MRS, according to HIV status. **Fig. S2.** NPA diagnostic yield using NAAT compared to children positive for MRS, according to age. **Fig. S3.** Scatterplot between the study microbiological confirmation rate and the diagnostic yield of NPA.

## Figures and Tables

**Table 1:** Study and patient characteristics

										No. of patients/total no. of patients (%)				
First author of study, year	Location	Country TB and HIV burden^a^	Study design	Participant recruitment timing	Clinical setting(s)	No. enrolled	Clinical features of cohort	Exclusion criteria	Median age (IQR) *(months)*	Female gender	Previous TB history	HIV positive	MRS +ve^c^	CRS +ve^d^
Franchi, 1998 [11]	Peru	MDR TB high burden	Cohort	Prospective	Hospital	64	Presumed pulmonary TB from clinical and epidemiological information	AIDS or previous TB Rx	85^b^ (NR)	NR	0/64	0/64 (0)	26/64 (41)	NA
Hanrahan, 2019 [24]	South Africa	TB + HIV/TB + MDR TB high burden	Cohort	Prospective	Primary healthcare	119	TB contact, cough, weight loss/failure to thrive, fever, lethargy	Received Rx for TB in past 3 months, the presence of danger signs (fever > 39 °C, severe respiratory distress, reduced consciousness) > 10 years of age	21 (12–43)	56/119 (47)	5/119 (4)	21/119 (18)	4/119 (3)	104/119 (87)
Marcy, 2016 [26]	Burkina Faso, Cameroon, Cambodia, Vietnam	TB high burden, TB/HIV high burden, TB + MDR TB high burden, not high-burden country	Cohort	Prospective	Hospital	281	Cough, failure to thrive, CXR abnormality, fever, failure of broad spectrum abx	Received Rx for TB in past 2 years	86 (49-NR)	132/272 (49)	49/272 (18)	272/272 (100)	35/272 (13)	246/272 (90)
Oberhelman, 2015 [27]	Peru	MDR TB high burden	Case control	Prospective	Hospital	290	TB contact, cough, CXR abnormality, TST/IGRA +ve, Stegen-Toledo score > 4 or > 3 if HIV positive	Not specified	NR	145/290 (50)	Unknown	81/290 (28)	23/290 (8)	NA
Owens, 2007 [28]	Uganda	TB + HIV/TB high burden	Cohort	Prospective	Hospital	96	Cough, weight loss, severe malnutrition, CXR abnormality, TST/IGRA +ve, failure of abx	HIV +ve and stable with CXR changes of bilateral symmetrical hilar opacities with diffuse parenchymal infiltration suggestive of LIP	48 (NR)	37/94 (39)	Unknown	44/94 (47)	24/94 (26)	NA
Song, 2021 [23]	Kenya	TB + HIV/TB high burden	Cohort	Prospective	Hospital, household contact tracing	300	Cough, weight loss/failure to thrive, CXR abnormality, fever, large persistent cervical LN, failure of abx	On TB Rx or IPT or if received Rx for TB in past year or IPT in past 6 months	24 (12–43)	151/300 (50)	Unknown	73/300 (24)	31/300 (10)	NA
Zar, 2012 [29]	South Africa	TB + HIV/TB + MDR TB high burden	Cohort	Prospective	Hospital	674	TB contact, cough, weight loss/failure to thrive, CXR abnormality TST/IGRA +ve	TB Rx for > 72 h did not live in Cape Town, IS or NPA sample not obtained	19 (11–38)	241/535 (45)	56/535 (10)	117/535 (22)	99/535 (19)	343/535 (64)
Zar, 2013 [25]	South Africa	TB + HIV/TB + MDR TB high burden	Cohort	Prospective	Primary healthcare	415	TB contact, cough, weight loss/failure to thrive, CXR abnormality TST/IGRA +ve	TB Rx for > 72 h, IS or NPA sample not obtained	38 (21–57)	203/384 (53)	42/384 (11)	31/384 (8)	42/384 (11)	197/384 (51)
Zar, 2019 [30]	South Africa	TB + HIV/TB + MDR TB high burden	Cohort	Prospective	Hospital	195	TB contact, cough, weight loss/failure to thrive, CXR abnormality TST/IGRA +ve	TB Rx for > 72 h did not live in Cape Town, IS or NPA sample not obtained	23 (14–47)	NR	15/195 (8)	32/195 (16)	45/195 (23)	144/195 (74)

Abbreviations: *CRS* composite reference standard, *GA* gastric aspirate, *IGRA* interferon gamma release assay, *IS* induced sputum, *IQR* interquartile range, *MRS* microbiological reference standard, *MDR-TB* multidrug-resistant TB, *NPA* nasopharyngeal aspirate, *NA* not applicable, *NR* not reported, *Rx* treatment, *TB* tuberculosis, *TST* tuberculin skin test

a As defined by the WHO [22]

b Mean reported, median not available

c Microbiologically confirmed TB by mycobacterial culture and/or a WHO-endorsed NAAT from NPA, GA, IS stool, string, and/or sputum specimens

d Microbiologically confirmed TB or with clinical, radiological, or immunologic features suggestive of TB but negative microbiological testing

**Table 2:** Summary of NPA collection, testing, and processing across studies

First author of study, year	Test(s) conducted on NPA	Fresh or frozen NPA samples	Duration of fasting prior to NPA	Saline drops during collection of NPA (no. of drops or volume)	Method for collection	Processing method of NPA	Adverse events reported	Number (%) of children with NPA collected successfully	Number (%) of contaminated NPA cultures	Number (%) of uninterpretable^a^ NPA NAAT results
Franchi, 1998 [11]	NAAT-PCR targeting DNA seq IS6110 Solid culture — LJ, 7H11 Liquid culture — MGIT 960	Fresh	NR	NR	NR	NR	No	64/64 (100%)	NR	NR
Hanrahan, 2019 [24]	NAAT-Xpert Liquid culture — MGIT 960	Fresh	2 h	Yes (4 drops)	Suction using a sterile catheter with a mucus trap	NALC-NaOH	Yes (low respiratory rate, *n* = 1)	101/105 (96%)	31/184 (17%)^b^	0/186^b^
Marcy, 2016 [26]	NAAT-Xpert Solid culture — LJ Liquid culture — MGIT 960	Fresh and frozen	Overnight	No	Mucus aspirator connected to a suction device	NALC-NaOH	No	268/272 (99%)	NR	13/272 (3%)^c^
Oberhelman, 2015 [27]	NAAT-PCR N2 heminested IS6110 Solid culture: LJ Liquid culture — MODS	Fresh	NR	Yes (5 ml)	Aspiration through a nasopharyngeal tube with an electrical suction device or handheld aspirator	NALC-NaOH	No	NR	NR	NR
Owens, 2007 [28]	NAAT-RealArt (commercially available real-time PCR assay) Solid culture — LJ	Fresh and frozen	NR	NR	Mechanical aspiration of secretions using a catheter	NALC-NaOH	Yes (emotional distress and epistaxis, *n* = 1)	95/96 (99%)	NR	NR
Song, 2021 [23]	NAAT-Xpert Liquid culture — MGIT 960	Fresh	Duration NR but collected at night before early morning and first meal of day	Yes (4 drops)	Suction using a 7- or 8-French tube connected to a mucus trap	NALC-NaOH	No	NR	NR	NR
Zar, 2012 [29]	NAAT-Xpert Liquid culture — MGIT 960	Fresh	2–3 h	Yes (4 drops)	Suction using a sterile catheter with a mucus trap	NALC-NaOH	No	NR	NR	At least 1/415^c^
Zar, 2013 [25]	NAAT-Xpert	Fresh	2–3 h	Yes (4 drops)	Suction using a sterile catheter with a mucus trap	NALC-NaOH	No	NR	NA	NR
Zar, 2019 [30]	NAAT-Xpert Ultra	Frozen	2–3 h	Yes (4 drops)	Suction using a sterile catheter with a mucus trap	NALC-NaOH	No	NR	NA	NR

Abbreviations: *LJ* Löwenstein–Jensen, *MGIT* mycobacteria growth indicator tube, *MODS* microscopic observation drug susceptibility, *NAAT* nucleic acid amplification test, *NPA* nasopharyngeal aspirate, *NALC-NaOH* N-acetyl-l-cysteine-sodium hydroxide, *NA* not applicable, *NR* not reported, *PCR* polymerase chain reaction

a Uninterpretable includes error, invalid, indeterminate, no result

b Denominator refers to number of NPA specimens collected in study

c Denominator refers to number of children in study

## Data Availability

All data on diagnostic yield analyzed during the current study are available in the main text or supplementary material.

## Abbreviations

CRS: Composite reference standard
GA: Gastric aspirate
MGIT: Mycobacteria Growth Indicator Tube
MODS: Microscopic observation drug susceptibility
MRS: Microbiological reference standard
Mtb: Mycobacterium tuberculosis
NAAT: Nucleic acid amplification test
NPA: Nasopharyngeal aspirate
PTB: Pulmonary tuberculosis
QUADAS-2: Quality assessment of diagnostic accuracy studies-2
ROB: Risk of bias
TB: Tuberculosis
WHO: World Health Organization

## References

[1] The World Health Organization. Global tuberculosis report 2022. Geneva: The WHO; 2022.

[2] Yerramsetti S, Cohen T, Atun R, Menzies NA. Global estimates of paediatric tuberculosis incidence in 2013–19: a mathematical modelling analysis. Lancet Glob Health. 2022;10(2):e207–15. 34895517 10.1016/S2214-109X(21)00462-9 PMC8800006

[3] Graham SM, Sismanidis C, Menzies HJ, Marais BJ, Detjen AK, Black RE. Importance of tuberculosis control to address child survival. Lancet. 2014;383(9928):1605–7. 24671079 10.1016/S0140-6736(14)60420-7 PMC5503686

[4] Perez-Velez CM, Roya-Pabon CL, Marais BJ. A systematic approach to diagnosing intra-thoracic tuberculosis in children. J Infect. 2017;74(Suppl 1):S74–83. 28646966 10.1016/S0163-4453(17)30195-0

[5] Dodd PJ, Yuen CM, Sismanidis C, Seddon JA, Jenkins HE. The global burden of tuberculosis mortality in children: a mathematical modelling study. Lancet Glob Health. 2017;5(9):e898–906. 28807188 10.1016/S2214-109X(17)30289-9 PMC5556253

[6] The World Health Organization. WHO consolidated guidelines on tuberculosis. Module 5: management of tuberculosis in children and adolescents. Geneva: The WHO; 2022. 35404556

[7] Wobudeya E, Bonnet M, Walters EG, Nabeta P, Song R, Murithi W, Diagnostic advances in childhood tuberculosis-improving specimen collection and yield of microbiological diagnosis for intrathoracic tuberculosis. Pathogens. 2022;11(4):389. 35456064 10.3390/pathogens11040389 PMC9025862

[8] Jensen PA, Lambert LA, Iademarco MF, Ridzon R. Guidelines for preventing the transmission of Mycobacterium tuberculosis in health-care settings. Atlanta: CDC; 2005. 16382216

[9] Reid MJ, Saito S, Fayorsey R, Carter RJ, Abrams EJ. Assessing capacity for diagnosing tuberculosis in children in sub-Saharan African HIV care settings. Int J Tuberc Lung Dis. 2012;16(7):924–7. 22583761 10.5588/ijtld.11.0816 PMC5920677

[10] The World Health Organization. Practical manual of processing stool samples for diagnosis of childhood TB. Geneva: The WHO; 2022.

[11] Franchi LM, Cama RI, Gilman RH, Montenegro-James S, Sheen P. Detection of Mycobacterium tuberculosis in nasopharyngeal aspirate samples in children. Lancet. 1998;352(9141):1681–2. 10.1016/s0140-6736(05)61454-7 9853450

[12] Marcy O, Wobudeya E, Font H, Vessière A, Chabala C, Khosa C, TB-Speed Pneumonia Study Group. Effect of systematic tuberculosis detection on mortality in young children with severe pneumonia in countries with high incidence of tuberculosis: a stepped-wedge cluster-randomised trial. Lancet Infect Dis. 2023;23(3):341–51. 10.1016/S1473-3099(22)00668-5. Epub 2022 Nov 14. 36395782

[13] Moher D, Liberati A, Tetzlaff J, Altman DG, Group P. Preferred Reporting Items for Systematic Reviews and Meta-Analyses: the PRISMA statement. J Clin Epidemiol. 2009;62(10):1006–12. 19631508 10.1016/j.jclinepi.2009.06.005

[14] Whiting PF, Rutjes AW, Westwood ME, Mallett S, Deeks JJ, Reitsma JB, QUADAS-2: a revised tool for the quality assessment of diagnostic accuracy studies. Ann Intern Med. 2011;155(8):529–36. 22007046 10.7326/0003-4819-155-8-201110180-00009

[15] Graham SM, Cuevas LE, Jean-Philippe P, Browning R, Casenghi M, Detjen AK, Clinical case definitions for classification of intrathoracic tuberculosis in children: an update. Clin Infect Dis. 2015;61Suppl 3:S179–87. 10.1093/cid/civ581 PMC4583568 26409281

[16] The World Health Organization. WHO operational handbook on tuberculosis. Module 3: Diagnosis - rapid diagnostics for tuberculosis detection. update. Geneva: The WHO; 2021. p. 2021.

[17] Viechtbauer W. Conducting meta-analyses in R with the metafor package. J Stat Softw. 2010;36(3):1–48.

[18] Balduzzi SRG, Schwarzer G. How to perform a meta-analysis with R: a practical tutorial. Evid Based Ment Health. 2019;22:153–60. 31563865 10.1136/ebmental-2019-300117 PMC10231495

[19] Oberhelman RA, Soto-Castellares G, Caviedes L, Castillo ME, Kissinger P, Moore DA, Improved recovery of Mycobacterium tuberculosis from children using the microscopic observation drug susceptibility method. Pediatrics. 2006;118(1):e100–6. 16751616 10.1542/peds.2005-2623 PMC7617044

[20] Oberhelman RA, Soto-Castellares G, Gilman RH, Caviedes L, Castillo ME, Kolevic L, Diagnostic approaches for paediatric tuberculosis by use of different specimen types, culture methods, and PCR: a prospective case-control study. Lancet Infect Dis. 2010;10(9):612–20. 20656559 10.1016/S1473-3099(10)70141-9 PMC2975578

[21] Walters E, van der Zalm MM, Demers AM, Whitelaw A, Palmer M, Bosch C, Specimen pooling as a diagnostic strategy for microbiologic confirmation in children with intrathoracic tuberculosis. Pediatr Infect Dis J. 2019;38(6):e128–31. 30418355 10.1097/INF.0000000000002240 PMC6509014

[22] The World Health Organization. WHO global lists of high burden countries for tuberculosis (TB), TB/HIV and multidrug/rifampicin-resistant TB (MDR/RR-TB), 2021–2025. Geneva: WHO; 2021.

[23] Song R, Click ES, McCarthy KD, Heilig CM, McHembere W, Smith JP, Sensitive and feasible specimen collection and testing strategies for diagnosing tuberculosis in young children. JAMA Pediatr. 2021;175(5):e206069. 33616611 10.1001/jamapediatrics.2020.6069 PMC7900937

[24] Hanrahan CF, Dansey H, Mutunga L, France H, Omar SV, Ismail N, Diagnostic strategies for childhood tuberculosis in the context of primary care in a high burden setting: the value of alternative sampling methods. Paediatr Int Child Health. 2019;39(2):88–94. 30378470 10.1080/20469047.2018.1533321

[25] Zar HJ, Workman L, Isaacs W, Dheda K, Zemanay W, Nicol MP. Rapid diagnosis of pulmonary tuberculosis in African children in a primary care setting by use of Xpert MTB/RIF on respiratory specimens: a prospective study. Lancet Glob Health. 2013;1(2):e97–104. 25104164 10.1016/S2214-109X(13)70036-6

[26] Marcy O, Ung V, Goyet S, Borand L, Msellati P, Tejiokem M, Performance of Xpert MTB/RIF and alternative specimen collection methods for the diagnosis of tuberculosis in HIV-infected children. Clin Infect Dis. 2016;62(9):1161–8. 26908804 10.1093/cid/ciw036

[27] Oberhelman RA, Soto-Castellares G, Gilman RH, Castillo ME, Kolevic L, Delpino T, A controlled study of tuberculosis diagnosis in HIV-infected and uninfected children in Peru. PLoS ONE. 2015;10(4):e0120915. 25927526 10.1371/journal.pone.0120915 PMC4416048

[28] Owens S, Abdel-Rahman IE, Balyejusa S, Musoke P, Cooke RP, Parry CM, Nasopharyngeal aspiration for diagnosis of pulmonary tuberculosis. Arch Dis Child. 2007;92(8):693–6. 17185437 10.1136/adc.2006.108308 PMC2083896

[29] Zar HJ, Workman L, Isaacs W, Munro J, Black F, Eley B, Rapid molecular diagnosis of pulmonary tuberculosis in children using nasopharyngeal specimens. Clin Infect Dis. 2012;55(8):1088–95. 22752518 10.1093/cid/cis598 PMC3529610

[30] Zar HJ, Workman LJ, Prins M, Bateman LJ, Mbhele SP, Whitman CB, Tuberculosis diagnosis in children using Xpert Ultra on different respiratory specimens. Am J Respir Crit Care Med. 2019;200(12):1531–8. 31381861 10.1164/rccm.201904-0772OC PMC6909828

[31] Srisuwanvilai LO, Monkongdee P, Podewils LJ, Ngamlert K, Pobkeeree V, Puripokai P, Performance of the BACTEC MGIT 960 compared with solid media for detection of Mycobacterium in Bangkok. Thailand Diagn Microbiol Infect Dis. 2008;61(4):402–7. 18440177 10.1016/j.diagmicrobio.2008.02.015

[32] Chihota VN, Grant AD, Fielding K, Ndibongo B, van Zyl A, Muirhead D, Liquid vs. solid culture for tuberculosis: performance and cost in a resource-constrained setting. Int J Tuberc Lung Dis. 2010;14(8):1024–31. 20626948

[33] Eisenach K, Demers AM, Jones F. Mycobacteriology laboratory sourcebook for harmonization and support of tuberculosis (TB) clinical trials, Version 1. Tucson: Critical Path to TB Drug Regimens (CPTR) Initiative; 2018.

[34] von Hippel PT. The heterogeneity statistic I(2) can be biased in small meta-analyses. BMC Med Res Methodol. 2015;15:35. 25880989 10.1186/s12874-015-0024-z PMC4410499

[35] The World Health Organization. WHO operational handbook on tuberculosis. Module 5: Management of tuberculosis in children and adolescents. Geneva: The WHO; 2022. 35404556

[36] Marais BJ, Hesseling AC, Gie RP, Schaaf HS, Enarson DA, Beyers N. The bacteriologic yield in children with intrathoracic tuberculosis. Clin Infect Dis. 2006;42(8):e69–71. 16575719 10.1086/502652

[37] Seid G, Alemu A, Tsedalu T, Dagne B. Value of urine-based lipoarabi-nomannan (LAM) antigen tests for diagnosing tuberculosis in children: systematic review and meta-analysis. IJID Reg. 2022;4:97–104. 35880002 10.1016/j.ijregi.2022.06.004 PMC9307507

[38] Kay AW, Ness T, Verkuijl SE, Viney K, Brands A, Masini T, Xpert MTB/RIF Ultra assay for tuberculosis disease and rifampicin resistance in children. Cochrane Database Syst Rev. 2022;9(9):CD013359. 36065889 10.1002/14651858.CD013359.pub3 PMC9446385

[39] MacLean E, Sulis G, Denkinger CM, Johnston JC, Pai M, Ahmad Khan F. Diagnostic Accuracy of Stool Xpert MTB/RIF for detection of pulmonary tuberculosis in children: a systematic review and meta-analysis. J Clin Microbiol. 2019;57(6):e02057–18. 30944200 10.1128/JCM.02057-18 PMC6535592

[40] Bhatta B, Vessière A, Borand L, Moh R, Khosa C, Chabala C, Acceptability of nasopharyngeal aspirate and stool for TB diagnosis in children with severe pneumonia: parents’ and healthcare workers’ perspective. Proceedings of the 52nd World Conference on Lung Health of the International Union Against Tuberculosis and Lung Disease (The Union); 19th-22 October 2021; Virtual. 2021.

[41] Spijker RDJ, Glanville J, Eisinga A. Chapter 6: Searching for and selecting studies. In: Deeks JJBP, Leeflang MM, Takwoingi Y, editors. Cochrane Handbook for Systematic Reviews of Diagnostic Test Accuracy Version 2. 2 ed. London: Cochrane; 2022.

[42] Parsons LM, Somoskovi A, Gutierrez C, Lee E, Paramasivan CN, Abimiku A, Laboratory diagnosis of tuberculosis in resource-poor countries: challenges and opportunities. Clin Microbiol Rev. 2011;24(2):314–50. 21482728 10.1128/CMR.00059-10 PMC3122496

[43] The Centers for Disease Control and Prevention. interim guidelines for collecting and handling of clinical specimens for COVID-19 testing Atlanta. 2022. Available from: https://www.cdc.gov/coronavirus/2019-ncov/lab/guidelines-clinical-specimens.html#handling-specimens-safely. Accessed 4 May 2023.

[44] Association of paediatric chartered physiotherapists. Guidelines for nasopharyngeal suction of a child or young adult. London. 2015. https://apcp.csp.org.uk/system/files/guidelines_for_nasopharyngeal_suction_0_1.pdf.Accessed 4 May 2023.

[45] Paediatric TB Operational and Sustainability Expertise Exchange (POSEE Taskforce). Summary guidance for microbiological and clinical diagnosis of pulmonary tuberculosis among children. Geneva: Stop TB Partnership. 2021. https://www.pedaids.org/wp-content/uploads/2021/06/POSEE-Info-Note_Pediatric-TB-diagnosis_Final_17.6.2021.pdf. Accessed 4 May 2023.

