# Supplementary material for: The diagnostic yield of nasopharyngeal aspirate for pediatric pulmonary tuberculosis: a systematic review and meta-analysis
Source: BMC Glob Public Health. Author manuscript; Available in PMC 2024 Apr 16. (PMC11019899; doi:10.1186/s44263-023-00018-1)
Supplement: Search strategy for systematic review and meta-analysis for each database. — Additional file 2. Search strategy for systematic review and meta-analysis for each database. [file NIHMS1980703-supplement-Search_strategy_for_systematic_review_and_meta-analysis_for_each_database_.docx]

# **Additional file 2: Search strategy for systematic review and meta-analysis for each database**

In line with the *Cochrane Handbook for Systematic Reviews of Diagnostic Test Accuracy,^1^* to maximise sensitivity, the search strategy focused on text words and database subject headings related to the index specimen (nasopharyngeal aspirate) and the target condition (tuberculosis) only.

**Search strategy used for PubMed**

(("NPA"[Title/Abstract] OR "Nasopharynx"[Title/Abstract] OR "Nasopharyngeal"[Title/Abstract] OR "nasal"[Title/Abstract] OR "nose"[MeSH Terms] OR "Nasopharynx"[MeSH Terms]) AND ("tuberculos*"[Title/Abstract] OR "TB"[Title/Abstract] OR "MTB"[Title/Abstract] OR "mycobacterium tuberculosis"[MeSH Terms] OR "tuberculosis"[MeSH Terms]))

NOT ("animals"[MeSH Terms] NOT "humans"[MeSH Terms])

No other filters were used.

**Search strategy used for EMBASE**

mycobacterium tuberculosis/ OR tuberculosis/ OR lung tuberculosis/ OR (tuberculos*).mp. OR MTB.mp. OR Tb.mp.

nasopharyngeal aspiration/ OR nose/ OR nasopharynx/  OR (nas* adj3 aspirat*).mp. OR (nas* adj3 swab*).mp. OR NPA.mp.

(exp animals/ or nonhuman/) not human/

Combine above with AND

No other filters were used.

**Search strategy used for Cochrane Library**

(tuberculos*):ti,ab,kw OR (TB):ti,ab,kw OR (MTB):ti,ab,kw OR [Tuberculosis] MeSH descriptor explode all trees

(NPA):ti,ab,kw OR (nas* NEAR/3 aspirat*):ti,ab,kw OR (nas* NEAR/3 swab*):ti,ab,kw OR MeSH descriptor [Nose] explode all trees OR MeSH descriptor [Nasopharnx] explode all trees

Combine above with AND

No other filters were used.

**Search strategy used for trial registers - ClinicalTrials.gov and WHO International Clinical Trials Registry Platform**

nasopharyngeal OR NPA

Tuberculosis OR TB

Combine above with AND

No other filters were used.

1. Spijker R DJ, Glanville J, Eisinga A. . Chapter 6: Searching for and selecting studies. . In: Deeks JJ BP, Leeflang MM, Takwoingi Y, editor(s). editor. Cochrane Handbook for Systematic Reviews of Diagnostic Test Accuracy Version 2. 2 ed. London: Cochrane; 2022.
