# Supplementary material for: The diagnostic yield of nasopharyngeal aspirate for pediatric pulmonary tuberculosis: a systematic review and meta-analysis
Source: BMC Glob Public Health. Author manuscript; Available in PMC 2024 Apr 16. (PMC11019899; doi:10.1186/s44263-023-00018-1)
Supplement: Adapted QUADAS-2 tool. — Additional file 3. Adapted QUADAS-2 tool. [file NIHMS1980703-supplement-Adapted_QUADAS-2_tool_.docx]

# **Additional file 3: Adapted QUADAS-2 Tool**

**DOMAIN 1: PATIENT SELECTION**

**Risk of bias: could the selection of patients have introduced bias?**

*Signaling question 1: Was a consecutive or random sample of patients enrolled?*

Yes/No/Unclear

In studies where it is not explicitly stated “all patients” or “consecutive”, we will answer “Unclear.”

*Signaling question 2: Was a case-control design avoided?*

Yes/No/Unclear

*Signaling question 3: Did the study avoid inappropriate exclusions?*

Yes/No/Unclear

Inappropriate exclusions would include very sick or malnourished children. Inappropriate inclusions would be including those confirmed to have TB based on prior diagnostic testing.

**Applicability: are there concerns that the included patients and setting do not match the review question?**

Studies in which the following are observed raises high concerns regarding applicability.

- if any enrolled children did not present with symptoms and signs suggestive of TB, including asymptomatic contacts, since they do not routinely undergo microbiological specimen collection in high-burden settings.

-studies that compared cases to healthy controls, which are not reflective of clinical practice.

**DOMAIN 2: INDEX TEST**

**Risk of bias: could the conduct or interpretation of the index test have introduced bias?**

*Signaling question 1: Were the index test results interpreted without knowledge of the results of the reference standard?*

Yes/No/Unclear

For studies which used tests on index nasopharyngeal (NPA) specimens that are automatically generated with categorical outcomes and subjective interpretation of test results is not expected, for example Xpert MTB/RIF (Xpert), Xpert MTB/RIF Ultra (Ultra) and Mycobacteria Growth Indicator Tube (MGIT) culture, we will say “Yes”. For tests for which subjective interpretation is possible, for e.g., in-house PCR tests, solid Löwenstein–Jensen (LJ) culture, or smear microscopy we will answer “No” unless blinding was explicitly stated.

*Signaling question 2: If a threshold was used, was it pre-specified?*

Yes/No/Unclear

The threshold is pre-specified for Xpert, Ultra and culture so for these tests, we will say “Yes”.

**Applicability: Are there concerns that the index test, its conduct, or interpretation differ from the review question?**

Various protocols for NPA exist from international (the World Health Organisation^1^, Stop for TB^2^) or national bodies (Centers for Disease Control and Prevention^3^). However, they are not uniform. In order to answer, “low concern”, studies must have reported an appropriate method of mucus extraction for example use of mucus/aspiration/sputum trap, with suction. For processing, studies must have described maintenance of cold chain until laboratory testing and decontamination procedure for example, NALC-NaOH.

**DOMAIN 3: REFERENCE STANDARD**

**Risk of bias: Could the reference standard, its conduct, or its interpretation have introduced bias?**

*Signaling question 1: Is a microbiological reference standard (MRS) likely to correctly classify the target condition?*

Yes/No/Unclear

In light of the imperfect MRS for pediatric TB diagnosis and since it is established that evaluation of multiple specimens and tests can increase the diagnostic yield of confirming TB, we will answer “Yes” if the MRS included multiple different specimens to diagnose TB (i.e. two or more, not including NPA) collected by a standardized approach and utilized both culture and a WHO-endorsed nucleic acid amplification test (NAAT) which increases the likelihood of confirming TB. Otherwise, we will answer “No”.

*Signaling question 2: Were the reference standard results interpreted without knowledge of the results of the index test?*

Yes/No/Unclear

For studies which used tests on reference specimens that are automatically generated with categorical outcomes and subjective interpretation of test results is not expected, for example Xpert, Ultra and MGIT culture, we will say “Yes”. For tests for which subjective interpretation is possible, for e.g., solid LJ culture, we will answer “No” unless blinding was explicitly stated.

**Applicability: Are there concerns that the target condition as defined by the reference standard does not match the question?**

For studies that used a culture method in their MRS we will state “high concern” if authors do not mention some type of specification assay to distinguish MTB from other mycobacteria. We expect heterogeneity across composite reference standards (CRS) and variations in the interpretation of clinical information (even if the study refers to published international case definitions). Therefore, for studies that used CRS, we will say unclear.

**DOMAIN 4: FLOW AND TIMING**

**Risk of bias: Could the patient flow have introduced bias?**

*Signaling question 1: Was there an appropriate interval between index test and reference standard?*

Yes/No/Unclear

We will answer “Yes” if the NPA and other specimens were collected and tested at the same time. We will also answer “Yes” if the time interval was less than or equal to one week, as long as anti-tuberculous treatment was not given between. If not tested immediately, authors must also have commented on appropriate storage (e.g., below 4^o^C for the first 72 hours, and at -80^o^C onwards)

*Signaling question 2: Did all patients receive the same reference standard?*

Yes/No/Unclear

Since the microbiological reference standard can be composed of multiple different specimens, we will answer “Yes” if all children from whom an NPA was collected also had another reference specimen collected. We will answer “No” if collection/testing of specimens was based on clinical discretion as opposed to a consistent approach for all children. We will also answer “No” if NPA and the other reference specimens were tested with different diagnostic assays (e.g., NPA specimens were only tested with Xpert, whereas others were tested with culture).

*Signaling question 4: Were all patients included in the analysis?*

Yes/No/Unclear

We will answer No if the number of children enrolled in the study did not match those in the analyses for our 2x2 tables, whereby the loss of numbers to the analysis is likely to have introduced bias (e.g., <10%).

1. The World Health Organization. Module 5: management of tuberculosis in children and adolescents. WHO operational handbook on tuberculosis. Geneva: WHO; 2022.
2. Paediatric TB Operational and Sustainability Expertise Exchange (POSEE Taskforce). Summary guidance for Microbiological and Clinical Diagnosis of pulmonary tuberculosis among children. Geneva: Stop TB Partnership; 2021.
3. The Centers for Disease Control and Prevention. Interim Guidelines for Collecting and Handling of Clinical Specimens for COVID-19 Testing Atlanta2022

[Available from: <https://www.cdc.gov/coronavirus/2019-ncov/lab/guidelines-clinical-specimens.html#handling-specimens-safely>.]
