# Supplementary material for: The diagnostic yield of nasopharyngeal aspirate for pediatric pulmonary tuberculosis: a systematic review and meta-analysis
Source: BMC Glob Public Health. Author manuscript; Available in PMC 2024 Apr 16. (PMC11019899; doi:10.1186/s44263-023-00018-1)
Supplement: MRS as defined by our review and CRS definitions across the studies. — Additional file 4: Table S1. MRS as defined by our review and CRS definitions across the studies. [file NIHMS1980703-supplement-MRS_as_defined_by_our_review_and_CRS_definitions_across_the_studies_.docx]

# **Additional file 4**

**Table S1: MRS as defined by our review and CRS definitions across the studies**

This table summarizes the diagnostic tests and specimens making up the MRS as defined in our systematic review and the CRS definitions across the included studies

|  | **Microbiological Reference Standard*** | | **Composite Reference Standard definition** |
| --- | --- | --- | --- |
|  | **Diagnostic Test** | **Specimens**  **(max no.)** |  |
| **Franchi 1998** | Solid culture - LJ, and 7H11;  Liquid culture- MGIT 960 | NPA (1), GA (1) | NA |
| **Hanrahan 2019** | NAAT- Xpert;  Liquid culture- MGIT 960; | NPA (2), sputum, IS, GA, stool | Children positive for CRS if either microbiologically confirmed TB through the MRS or unconfirmed based on symptoms and signs, chest x-ray, contact history or immunologic evidence and treatment response (as per international case definition)^1^. |
| **Marcy 2016** | NAAT- Xpert;  Solid culture- LJ;  Liquid culture- MGIT 960; | NPA (1), sputum (3), GA (2), stool (1), string (1)* | Children positive for CRS if either microbiologically confirmed TB through the MRS or “probable”, or “possible” TB based on symptom and signs, chest x-ray, contact history or immunologic evidence, and treatment response (as per international case definition)^2^. |
| **Oberhelman 2015** | Solid culture: LJ;  Liquid culture- MODS | NPA (2), GA (2), stool (2) | NA |
| **Owens 2007** | Solid culture- LJ; | NPA (1), IS (1) | NA |
| **Song 2021** | NAAT- Xpert;  Liquid culture- MGIT 960; | NPA (2), IS (2) GA (2), stool (2), string (2) | NA |
| **Zar 2012** | NAAT- Xpert;  Liquid culture- MGIT 960; | NPA (2), IS (2) | Children positive for CRS if either microbiologically confirmed TB through the MRS or “possible TB” defined as all children in the study:  a. who did not have bacterial confirmation AND  b. who after not receiving TB treatment, did not have documented resolution of symptoms and signs at follow-up. |
| **Zar 2013** | NAAT- Xpert;  Liquid culture- MGIT 960; | NPA (2), IS (2) | Children positive for CRS if either microbiologically confirmed TB through the MRS or “possible TB” defined as all children in the study:  a. who did not have bacterial confirmation AND  b. who after not receiving TB treatment, did not have documented resolution of symptoms and signs at follow-up. |
| **Zar 2019** | NAAT- Xpert;  Liquid culture- MGIT 960; | NPA (2), IS (2) | Children positive for CRS if either microbiologically confirmed TB through a MRS or unconfirmed based on TB symptoms, chest x-ray, contact history or immunologic evidence and treatment response (as per international case definition)^1^. |

*MRS was defined as mycobacterial culture and/or a WHO-endorsed NAAT on any clinical specimen traditionally used to diagnose childhood PTB, as per published international case definitions for pediatric intrathoracic research^1^.

** In this study, gastric aspirates were taken for children aged <10 years and expectorated sputum samples were taken for children aged ≥10 years, nasopharyngeal aspirate and stool were taken for all children, and a string test if the child was aged ≥4 years.

Abbreviations: CRS: composite reference standard, GA: gastric aspirate, IS: induced sputum, LJ: Löwenstein–Jensen, MGIT: Mycobacteria Growth Indicator Tube, MODS: Microscopic observation drug susceptibility, MRS: microbiological reference standard, NAAT: nucleic acid amplification test, NPA: nasopharyngeal aspirate, PCR: polymerase chain reaction

1. Graham SM, Cuevas LE, Jean-Philippe P, Browning R, Casenghi M, Detjen AK, et al. Clinical Case Definitions for Classification of Intrathoracic Tuberculosis in Children: An Update. Clin Infect Dis. 2015;61Suppl 3:S179-87.
2. Graham SM, Ahmed T, Amanullah F, Browning R, Cardenas V, Casenghi M, et al. Evaluation of tuberculosis diagnostics in children: 1. Proposed clinical case definitions for classification of intrathoracic tuberculosis disease. Consensus from an expert panel. J Infect Dis. 2012;205 Suppl 2(Suppl 2):S199-208.
