# Supplementary material for: The diagnostic yield of nasopharyngeal aspirate for pediatric pulmonary tuberculosis: a systematic review and meta-analysis
Source: BMC Glob Public Health. Author manuscript; Available in PMC 2024 Apr 16. (PMC11019899; doi:10.1186/s44263-023-00018-1)
Supplement: Detailed summary of QUADAS-2 assessments for each study. — Additional file 5: Table S2. Detailed summary of QUADAS-2 assessments for each study. [file NIHMS1980703-supplement-Detailed_summary_of_QUADAS-2_assessments_for_each_study_.docx]

# **Additional file 5**

**Table S2: Detailed summary of QUADAS-2 assessments for each study**

|  | | Franchi, 1998 | Hanrahan, 2019 | Marcy, 2016 | Oberhelman, 2015 | Owens, 2007 | Song, 2021 | Zar, 2012 | Zar, 2013 | Zar, 2019 |
| --- | --- | --- | --- | --- | --- | --- | --- | --- | --- | --- |
| Domain: PATIENT SELECTION | | | | | | | | | | |
| *Signaling question 1* | Was a consecutive or random sample of patients enrolled? | Unclear | Unclear | Unclear | Unclear | Unclear | Yes | Yes | Yes | Yes |
| *Signaling question 2* | Was a case-control design avoided? | Yes | Yes | Yes | No | Yes | Yes | Yes | Yes | Yes |
| *Signaling question 3* | Did the study avoid inappropriate exclusions? | Yes | No-  excluded clinically unwell children and children above 10 years | Yes | Yes | Yes | Yes | Yes | Yes | Yes |
| ROB |  | Unclear | High | Unclear | High | Unclear | Low | Low | Low | Low |
| Applicability concerns | Are there concerns that the included patients do not match the review question? | Low | Low | Low | High- case control study including asymptomatic children with positive TST result | Low | Low | Low | Low | Low |
| Domain: INDEX TEST | | | | | | | | | | |
| *Signaling question 1* | Were the index test results interpreted without knowledge of the results of the reference standard? | No - used solid culture and in-house PCR without explicitly stating blinding | Yes | Yes | Yes | No- used solid culture and in-house PCR without explicitly stating blinding | Yes | Yes | Yes | Yes |
| *Signaling question 2* | If a threshold was used, was it prespecified? | No - used solid culture and in-house PCR | Yes | Yes | No- used solid culture and in-house PCR | No- used solid culture and in-house PCR | Yes | Yes | Yes | Yes |
| ROB |  | High | Low | Low | High | High | Low | Low | Low | Low |
| Applicability concerns | *Applicability: Are there concerns that the index test, its conduct, or interpretation differ from the review question?* | High- procedure for NPA collection or processing not described | Low | Low | Low | Unclear-procedure for NPA collection inadequately described | Low | Low | Low | Low |
| Domain: REFERENCE STANDARD | | | | | | | | | | |
| *Signaling question 1* | Is the microbiological reference standard likely to correctly classify the target condition? | No- only included one other type of specimen (excluding NPA) in the MRS and no WHO-endorsed NAAT | Yes | Yes | No- did not use a WHO-endorsed NAAT | No- only included one other type of specimen (excluding NPA) in the MRS and no WHO-endorsed NAAT | Yes | No- only included one other type of specimen (excluding NPA) in the MRS | No- only included one other type of specimen (excluding NPA) in the MRS | No- only included one other type of specimen (excluding NPA) in the MRS |
| *Signaling question 2* | Were the microbiological reference standard results interpreted without knowledge of the results of the index test? | No | Yes | Yes | Yes | No | Yes | Yes | Yes | Yes |
| ROB |  | High | Low | Low | High | High | Low | High | High | High |
| Applicability | *Are there concerns that the target condition as defined by the reference standard does not match the question?* | High- did not report MTB speciation methods | High- did not report MTB speciation methods | Unclear- used a CRS | High- did not report MTB speciation methods | High- did not report MTB speciation methods | Low | Unclear- used a CRS | Unclear- used a CRS | Unclear- used a CRS |
| Domain: FLOW & TIMING | | | | | | | | | | |
| *Signaling question 1* | Was there an appropriate interval between index test(s) and reference standard? | Yes | Unclear- not clear if TB treatment started before collecting some specimens | Yes | Yes | Yes | Yes | Yes | Yes | Yes |
| *Signaling question 2* | Did all patients receive the same microbiological reference standard? | Yes | Yes | No- children received different reference tests with differing sensitivities (MGIT or LJ culture) | Yes | Yes | Yes | Yes | Yes | Yes |
| *Signaling question 3* | Were all patients included in the analysis and if not, was it unlikely to have introduced bias. | Yes | Yes | Yes | Yes | Yes | Yes | No- 535 in the analyses compared to 674 enrolled (loss of 20%) | Yes | Yes |
| ROB |  | Low | Unclear | High | Low | Low | Low | High | Low | Low |

Abbreviations: CRS; composite reference standard, LJ: Löwenstein–Jensen, MGIT: Mycobacteria Growth Indicator Tube, MRS: microbiological reference standard, NAAT: nucleic acid amplification test, NPA: nasopharyngeal aspirate, MTB: mycobacterium tuberculosis, PCR: polymerase chain reaction, ROB: risk of bias, TST: tuberculin skin test
