# Supplementary material for: The diagnostic yield of nasopharyngeal aspirate for pediatric pulmonary tuberculosis: a systematic review and meta-analysis
Source: BMC Glob Public Health. Author manuscript; Available in PMC 2024 Apr 16. (PMC11019899; doi:10.1186/s44263-023-00018-1)
Supplement: Diagnostic yield for NPA culture and NPA NAAT compared to children positive for MRS. — Additional file 6: Table S3. Diagnostic yield for NPA culture and NPA NAAT compared to children positive for MRS. [file NIHMS1980703-supplement-Diagnostic_yield_for_NPA_culture_and_NPA_NAAT_compared_to_children_positive_for_MRS_.docx]

# **Additional file 6**

**Table S3: Diagnostic yield for NPA culture and NPA NAAT compared to children positive for MRS**

| **Study first author, year** | **No. of children positive for TB by NPA culture** | **No. of children positive for TB by NPA NAAT** | **Total no. of children positive by MRS*** | **Diagnostic yield of NPA culture (95% CI)** | **Diagnostic yield of NPA NAAT (95% CI)** |
| --- | --- | --- | --- | --- | --- |
| Franchi, 1998 | 19 | NA | 26 | 0.73 (0.52-0.88) | NA |
| Hanrahan, 2019 | 1 | 2 | 4 | 0.25 (0.01-0.81) | 0.50 (0.07-0.93) |
| Marcy, 2016 | 22 | 21 | 35 | 0.63 (0.45-0.79) | 0.60 (0.42-0.76) |
| Oberhelman, 2015 | 4 | NA | 23 | 0.17 (0.05-0.39) | NA |
| Owens, 2007 | 21 | NA | 24 | 0.88 (0.68-0.97) | NA |
| Song, 2021 | 21 | 14 | 31 | 0.68 (0.49-0.83) | 0.45 (0.27-0.64) |
| Zar, 2012 | 50 | 41 | 99 | 0.51 (0.40-0.61) | 0.41 (0.32-0.52) |
| Zar, 2013 | NA | 13 | 42 | NA | 0.31 (0.18-0.47) |
| Zar, 2019 | NA | 20 | 45 | NA | 0.44 (0.30-0.60) |

*MRS was defined as mycobacterial culture and/or a WHO-endorsed NAAT on any clinical specimen traditionally used to diagnose childhood PTB, as per international case definitions for pediatric intrathoracic research^1^.

Abbreviations: CI: confidence interval, MRS: microbiological reference standard, NAAT: nucleic acid amplification test, NPA: nasopharyngeal aspirate, TB: tuberculosis

1. Graham SM, Cuevas LE, Jean-Philippe P, Browning R, Casenghi M, Detjen AK, et al. Clinical Case Definitions for Classification of Intrathoracic Tuberculosis in Children: An Update. Clin Infect Dis. 2015;61Suppl 3:S179-87.
