# Supplementary material for: The diagnostic yield of nasopharyngeal aspirate for pediatric pulmonary tuberculosis: a systematic review and meta-analysis
Source: BMC Glob Public Health. Author manuscript; Available in PMC 2024 Apr 16. (PMC11019899; doi:10.1186/s44263-023-00018-1)
Supplement: Diagnostic yield for NPA culture and NPA NAAT compared to a modified MRS. — Additional file 7: Table S4. Diagnostic yield for NPA culture and NPA NAAT compared to a modified MRS. [file NIHMS1980703-supplement-Diagnostic_yield_for_NPA_culture_and_NPA_NAAT_compared_to_a_modified_MRS_.docx]

# **Additional file 7**

**Table S4: Diagnostic yield for NPA culture and NPA NAAT compared to a modified MRS**

| **Study first author, year** | **No. of children positive for TB with positive NPA culture** | **No. of children positive for TB with positive NPA NAAT** | **Total no. of children positive by modified MRS** | **Diagnostic yield of NPA culture (95% CI)** | **Diagnostic yield of NPA NAAT (95% CI)** |
| --- | --- | --- | --- | --- | --- |
| Franchi, 1998 | 17 | NA | 24 | 0.71 (0.49-0.87) | NA |
| Hanrahan, 2019 | 1 | 1 | 3 | 0.33 (0.01-0.91) | 0.33 (0.01-0.91) |
| Oberhelman, 2015 | 4 | NA | 23 | 0.17 (0.05-0.39) | NA |
| Owens, 2007 | 16 | NA | 19 | 0.84 (0.60-0.97) | NA |
| Song, 2021 | 18 | 13 | 28 | 0.64 (0.44-0.81) | 0.46 (0.28-0.66) |

This table summarizes the diagnostic yield for NPA culture and NAAT compared to children positive for a modified MRS, defined as mycobacterial culture and/or a WHO-endorsed NAAT on any clinical specimen traditionally used to diagnose childhood PTB, excluding NPA.

Abbreviations: CI: confidence interval, MRS: microbiological reference standard, NAAT: nucleic acid amplification test, NPA: nasopharyngeal aspirate, TB: tuberculosis
