# Supplementary material for: The diagnostic yield of nasopharyngeal aspirate for pediatric pulmonary tuberculosis: a systematic review and meta-analysis
Source: BMC Glob Public Health. Author manuscript; Available in PMC 2024 Apr 16. (PMC11019899; doi:10.1186/s44263-023-00018-1)
Supplement: Diagnostic yield for NPA culture and NPA NAAT compared to children positive for CRS. — Additional file 8: Table S5. Diagnostic yield for NPA culture and NPA NAAT compared to children positive for CRS. [file NIHMS1980703-supplement-Diagnostic_yield_for_NPA_culture_and_NPA_NAAT_compared_to_children_positive_for_CRS_.docx]

# **Additional file 8**

**Table S5: Diagnostic yield for NPA culture and NPA NAAT compared to children positive for CRS**

| **Study first author, year** | **No. of children positive for TB by NPA culture** | **No. of children positive for TB by NPA NAAT** | **Total no. of children positive by CRS*** | **Diagnostic yield of NPA culture (95% CI)** | **Diagnostic yield of NPA NAAT (95% CI)** |
| --- | --- | --- | --- | --- | --- |
| Hanrahan, 2019 | 1 | 2 | 104 | 0.01 (0-0.05) | 0.02 (0-0.07) |
| Marcy, 2016 | 22 | 21 | 246 | 0.09 (0.06-0.13) | 0.09 (0.05-0.13 |
| Zar, 2012 | 50 | 41 | 343 | 0.15(0.11-0.19) | 0.12 (0.09-0.16) |
| Zar, 2013 | NA | 13 | 197 | NA | 0.07 (0.04-0.11) |
| Zar, 2019 | NA | 20 | 144 | NA | 0.14 (0.09-0.21) |

*CRS were defined as reported in the original publications.

Abbreviations: CI: confidence interval, CRS: composite reference standard, NAAT: nucleic acid amplification test, NPA: nasopharyngeal aspirate, TB: tuberculosis
