# Supplementary material for: The diagnostic yield of nasopharyngeal aspirate for pediatric pulmonary tuberculosis: a systematic review and meta-analysis
Source: BMC Glob Public Health. Author manuscript; Available in PMC 2024 Apr 16. (PMC11019899; doi:10.1186/s44263-023-00018-1)
Supplement: Summary estimates for diagnostic yield from main and sensitivity analyses, compared to MRS. — Additional file 9: Table S6. Summary estimates for diagnostic yield from main and sensitivity analyses, compared to MRS. [file NIHMS1980703-supplement-Summary_estimates_for_diagnostic_yield_from_main_and_sensitivity_analyses__compared_to_MRS_.docx]

# **Additional file 9**

**Table S6: Summary estimates for diagnostic yield from main and sensitivity analyses, compared to MRS**

Main analyses refers to all included studies. Sensitivity analysis 1 refers only to studies with a low risk of bias for the reference standard domain of the QUADAS-2. assessment. Sensitivity analysis 2 refers only to studies with both culture and NAAT in the MRS denominator for the estimate of diagnostic yield.

|  | **NPA culture** | | | **NPA NAAT** | | |
| --- | --- | --- | --- | --- | --- | --- |
|  | **No of studies included**  **(total no. of children)** | **Pooled diagnostic yield (95% CI)** | **I^2^ statistic (%) (95% CI)** | **No of studies included**  **(total no. of children)** | **Pooled diagnostic yield (95% CI)** | **I^2^ statistic %**  **(95% CI)** |
| Main analyses | 7 (242) | 0.58 (0.42-0.73) | 77 (57-98) | 6 (256) | 0.44 (0.36-0.51) | 25 (0-88) |
| Sensitivity analyses 1 | 3 (70) | 0.63 (0.51-0.74) | 12 (0-99) | 3 (70) | 0.53 (0.41-0.64) | 0 (0-94) |
| Sensitivity analyses 2 | 4 (169) | 0.57 (0.46-0.68) | 39 (0-99) | 6 (256)* | 0.44 (0.36-0.51)* | 25 (0-88)* |

*Note: all studies in the main analysis for NPA NAAT included both culture and NAAT in the MRS so no study was excluded in sensitivity analyses 2

Abbreviations: CI: confidence interval, MRS: microbiological reference standard, NAAT: nucleic acid amplification test, NPA: nasopharyngeal aspirate
