# Supplementary material for: The diagnostic yield of nasopharyngeal aspirate for pediatric pulmonary tuberculosis: a systematic review and meta-analysis
Source: BMC Glob Public Health. Author manuscript; Available in PMC 2024 Apr 16. (PMC11019899; doi:10.1186/s44263-023-00018-1)
Supplement: Fig. S1. NPA diagnostic yield compared to children positive for MRS, according to HIV status. Fig. S2. NPA diagnostic yield using NAAT compared to children positive for MRS, according to age. Fig. S3. Scatterplot between the study microbiological confirmation rate and the diagnostic yield of NPA. — Additional file 10: Fig. S1. NPA diagnostic yield compared to children positive for MRS, according to HIV status. Fig. S2. NPA diagnostic yield using NAAT compared to children positive for MRS, according to age. Fig. S3. Scatterplot between the study microbiological confirmation rate and the diagnostic yield of NPA. [file NIHMS1980703-supplement-Fig__S1__NPA_diagnostic_yield_compared_to_children_positive_for_MRS__according_to_HIV_status__Fig__S2__NPA_diagnostic_yield_using_NAAT_compared_to_children_positive_for_MRS__according_to_age__Fig__S3__Scatterplot_betwe.docx]

# **Additional file 10**

**Figure S1: NPA diagnostic yield compared to children positive for MRS, according to HIV status**

A) NAAT in children with HIV

B) NAAT in HIV negative children

C) Culture in children living with HIV

D) Culture in HIV negative children

**
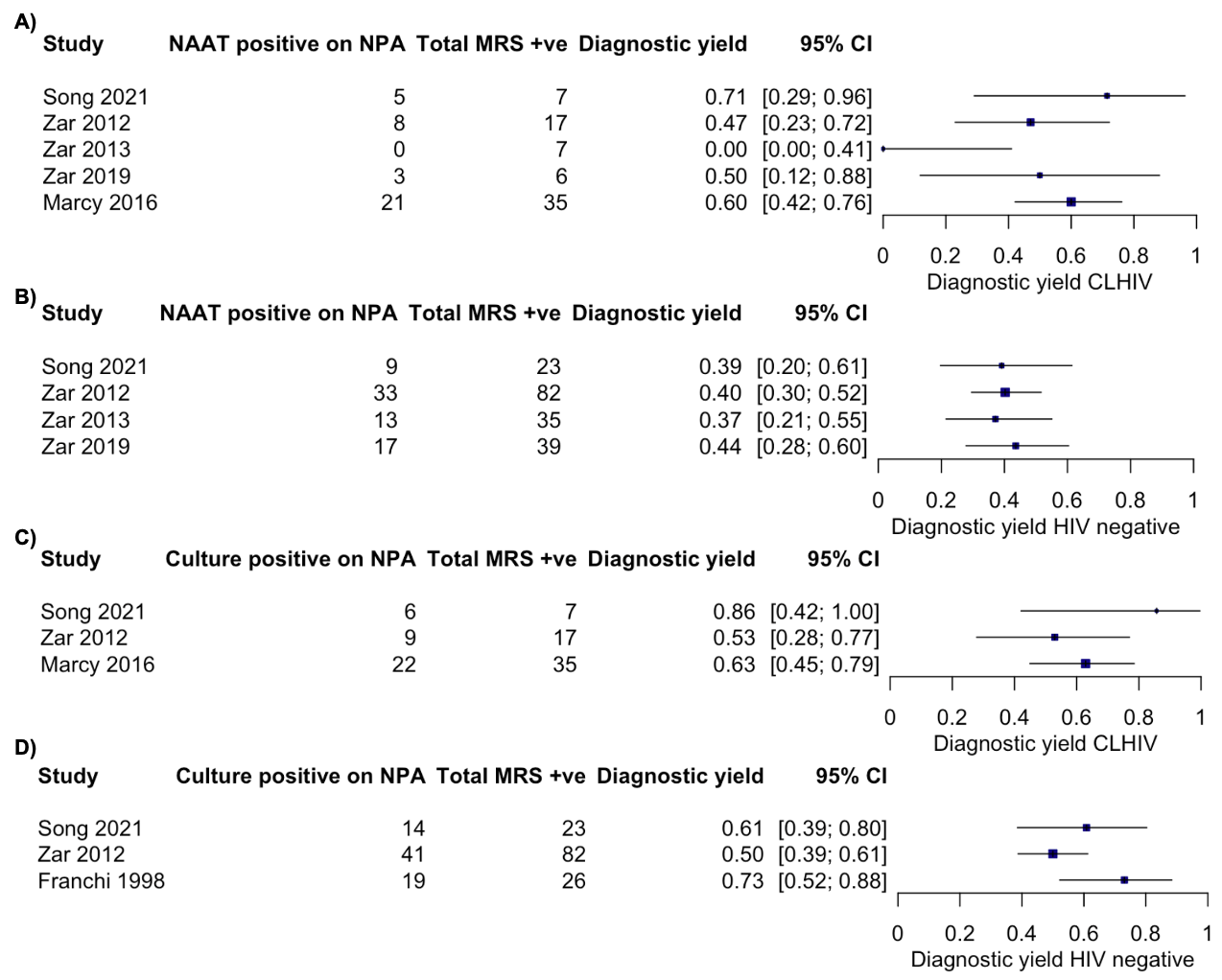
**

Abbreviations: CI: confidence interval, CLHIV: children living with HIV; MRS: microbiological reference standard, NAAT: nucleic acid amplification test, NPA: nasopharyngeal aspirate

**Figure S2: NPA diagnostic yield using NAAT compared to children positive for MRS, according to age**

A) in children younger than five years

B) in children five years or older

Data on yield stratified by age was only available for NAAT.

**
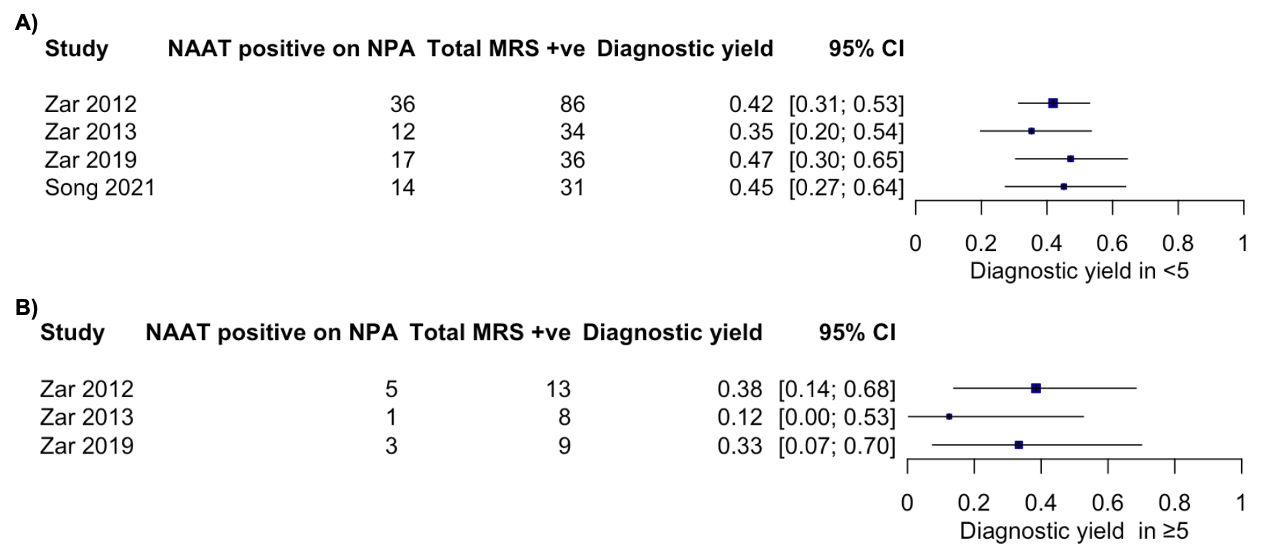
**

Abbreviations: CI: confidence interval, MRS: microbiological reference standard, NAAT: nucleic acid amplification test, NPA: nasopharyngeal aspirate

**Figure S3: Scatterplot between the study microbiological confirmation rate and the diagnostic yield of NPA**

1. Culture on NPA
2. NAAT on NPA

Included studies are represented by circles, with the size of the circle proportional to study weight (i.e., larger studies have a larger circle)

**
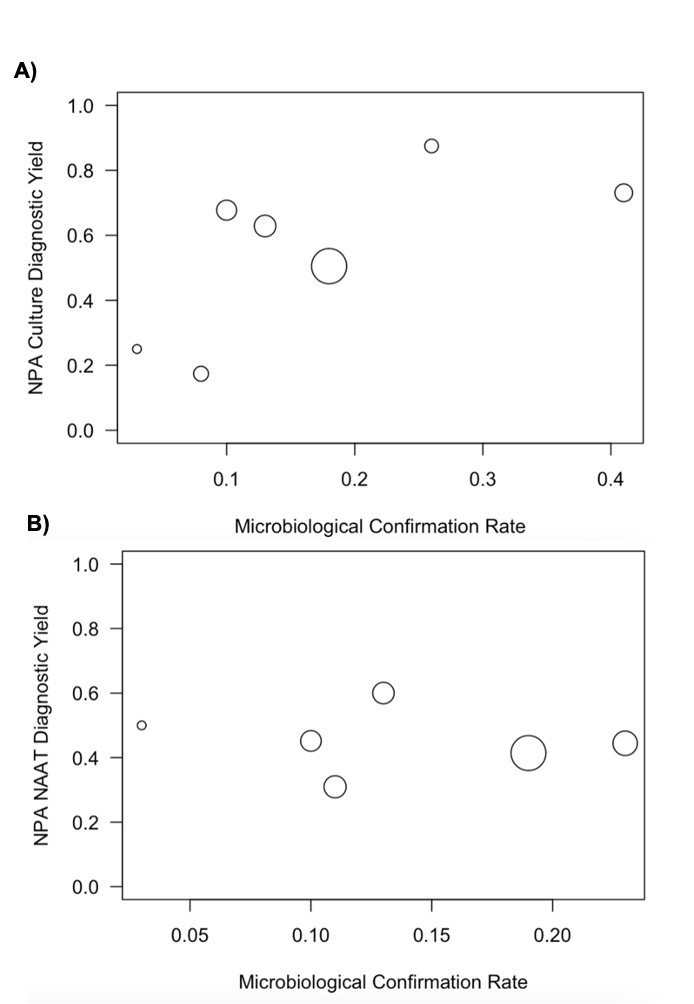
**

Abbreviations: NAAT: nucleic acid amplification test, NPA: nasopharyngeal aspirate
